# A case series of confirmed *Neisseria gonorrhoeae* native mitral valve infective endocarditis: presentation and management

**DOI:** 10.1093/ehjcr/ytag531

**Published:** 2026-07-11

**Authors:** Stefan Van Der Westhuizen, Johan Koen, Jan Steyn, Danielle Meintjes, Scott Lee-Jones, Jacques Janson, Anton Frans Doubell

**Affiliations:** Division of Cardiothoracic Surgery, Department of Surgical Sciences, Stellenbosch University and Tygerberg Hospital, Cape Town, South Africa; Division of Cardiothoracic Surgery, University of Toronto, Toronto, Canada; Division of Cardiology, Tygerberg Hospital, Cape Town, South Africa; Division of Internal Medicine, Department of Medicine, Stellenbosch University and Tygerberg Hospital, Cape Town, South Africa; Division of Infectious Diseases, Department of Medicine, Tygerberg Hospital and Stellenbosch University, Cape Town, South Africa; Division of Cardiothoracic Surgery, Department of Surgical Sciences, Stellenbosch University and Tygerberg Hospital, Cape Town, South Africa; Division of Cardiology, Department of Medicine, Faculty of Medicine and Health Sciences, University of Stellenbosch, Cape Town, South Africa

**Keywords:** Gonococcal endocarditis, DGI, Mitral valve endocarditis, BCNIE, Case series, Valve repair

## Abstract

**Background:**

Infective endocarditis (IE) arising as a complication of disseminated gonococcal infection (DGI) is uncommon. The condition most frequently involves the aortic or pulmonary valves, with 12 cases affecting the native mitral valve reported in the literature to date. We describe a rare occurrence of three patients presenting with native mitral valve gonococcal endocarditis.

**Case summary:**

Three unrelated patients presented to our unit over a 15-month period with DGI. The clinical presentations varied, and although sexually active, no recent prodromal history of DGI were reported, except for one patient who had non-specific complaints of tender joints potentially in keeping with gonococcal infection. Two patients underwent a surgical mitral valve replacement due to significant valve destruction, and one underwent mitral valve repair. Laboratory testing for organism identification required a multi-modal strategy in all patients. One patient demised as a complication of over-anticoagulation.

**Discussion:**

Diagnosis and treatment of patients presenting with IE should follow the 2023 ESC Guidelines on Infective Endocarditis. A multimodal strategy should be employed in identifying the organism, especially in the case of culture-negative endocarditis. Due to the extensive tissue destruction seen with gonococcal IE the patients mostly require valve replacement. However, valve repair must be considered/aimed for in our patients given the morbidity and mortality with warfarin therapy as highlighted by this case series. Local sensitivity profiles and resistance testing should be considered when treating DGI.

Learning points

- A high index of suspicion for *Neisseria gonorrhoeae* must be maintained in BCNIE despite absence of symptoms or signs.
- In conjunction with the 2023 ESC guideline surgical principles, mitral valve repair should be prioritized where achievable.
- Local antimicrobial susceptibility must dictate antibiotic choice in gonococcal endocarditis.

## Introduction

Infective endocarditis (IE) as a complication of disseminated gonococcal infection (DGI) is exceedingly rare; seen only in 1–2% of cases.^1^ Published prevalence data derive predominantly from 1950s–1970s literature, reflecting the higher disease burden before standardized antibiotic therapy. Sexually transmitted infections (STIs) caused by *Neisseria gonorrhoeae*, especially cephalosporin-resistant strains, have resurged across all demographics in countries such as the United States.^2^

Disseminated gonococcal infection endocarditis traditionally affects the aortic valve or root, occasionally the pulmonary valve; mitral involvement is rare.^3^ A PubMed Central search identified approximately 80 cases of DGI since 1939, with 12 involving the native mitral valve alone. We describe three patients with native mitral valve gonococcal endocarditis managed at our institution over a 15-month period, highlighting the variable presentation and the importance of further diagnostic testing as advocated by the 2023 Duke-ISCVID criteria.

## Summary figure

**Figure:**
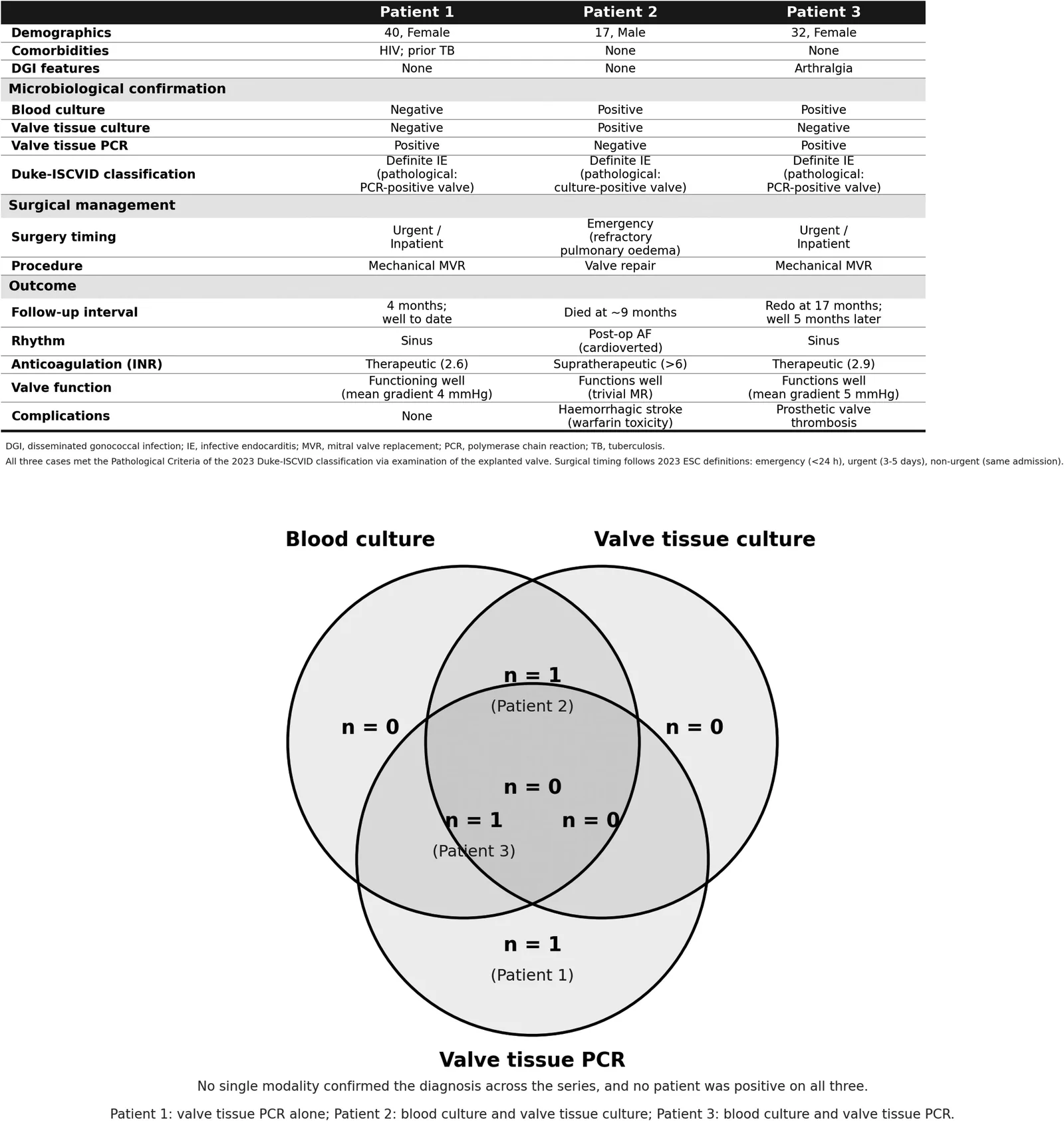


### Patient 1

A 40-year-old woman living with human immunodeficiency virus (HIV) presented to the emergency department with fever and persistent non-haemorrhagic diarrhoea. She was haemodynamically stable with mild dehydration. She had prior pulmonary tuberculosis (fully treated) and was established on antiretroviral therapy with an undetectable viral load and CD4 count of 1386.

The patient reported a 2-month history of fever, night sweats, and weight loss. Examination revealed left eye ptosis, right upper arm paresis, and a 4/6 apical systolic murmur. No local features of gonococcal infection were observed.

One blood culture set (comprising two aerobic and one anaerobic BacT/Alert FA Plus bottles, drawn from a single antecubital venepuncture site) was obtained; all bottles remained negative after 7 days of incubation. Empiric ceftriaxone 1 g intravenously twice daily was initiated, with doxycycline 100 mg orally twice daily added given the high local prevalence of Bartonella endocarditis. Serological testing for Bartonella subspecies was also obtained. Contrast computed tomography of the brain demonstrated a subacute to chronic left caudate nucleus infarction. A lumbar puncture was acellular and produced no bacterial growth. Stool culture showed no infective aetiology.

**Figure 1:**
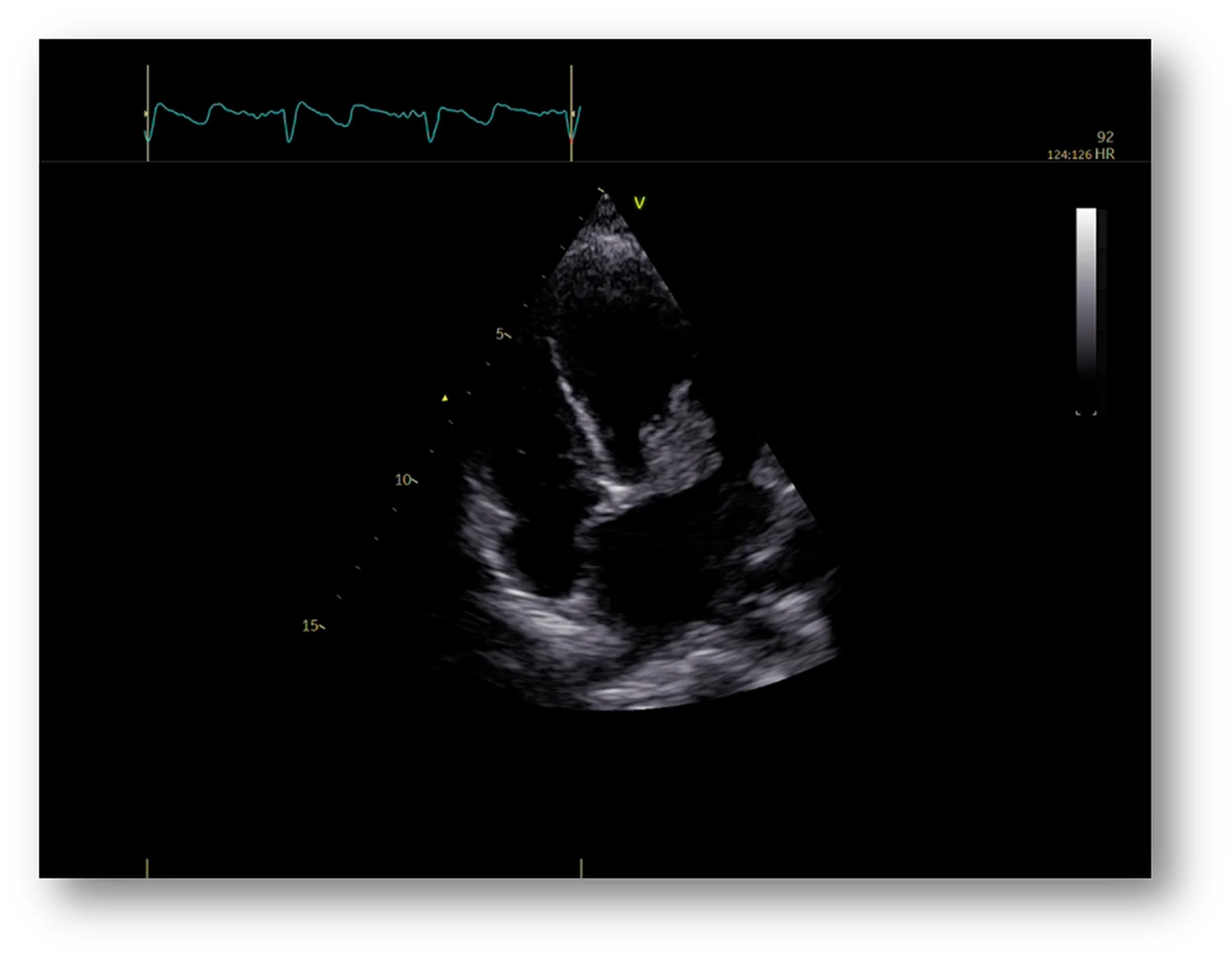
Apical four-chamber view demonstrating large vegetation on anterior mitral valve leaflet (Patient 1).

Transthoracic echocardiography demonstrated a large vegetations, up to 2 cm, on the A3 and P3 scallops extending towards the medial commissure, with acute severe mitral regurgitation (MR) (*Figure 1*).

The valve was not amenable to repair; a 29 mm St Jude Medical mechanical mitral valve replacement (MVR) was performed on an urgent inpatient basis 10 days after admission (reflecting surgical list availability), per the 2023 ESC indication for heart failure due to valvular regurgitation. The 16S ribosomal RNA polymerase chain reaction (PCR) on explanted valve tissue was positive for *N. gonorrhoeae*. Valve tissue culture was negative at 7 days, and all blood cultures remained negative throughout the admission. Histopathological features were in keeping with acute inflammation.

Doxycycline was stopped once Bartonella serology returned negative. Following surgery, targeted ceftriaxone 2 g intravenously daily (as 1 g twice daily) was continued for 6 weeks, and the left arm paresis improved with physiotherapy.

At 4 months she was NYHA class I, in sinus rhythm with a therapeutic INR and a well-functioning prosthesis, and has remained well since. Applying the 2023 Duke-ISCVID criteria, this was definite IE: a Pathological Criterion was met by identification of *N. gonorrhoeae* on PCR of the explanted valve, and a surgical Major Criterion by intra-operative confirmation of an infected valve (vegetation with leaflet destruction). The diagnostic basis for all three patients is shown in the Venn Summary.

### Patient 2

A previously well 17-year-old male presented with severe progressive exertional dyspnoea on a background of a 3-week history of malaise, fever, and night sweats. No history or clinical features of systemic gonococcal infection were noted.

On presentation he was in acute heart failure with pulmonary oedema, with a loud non-radiating apical pansystolic murmur.

**Figure 2:**
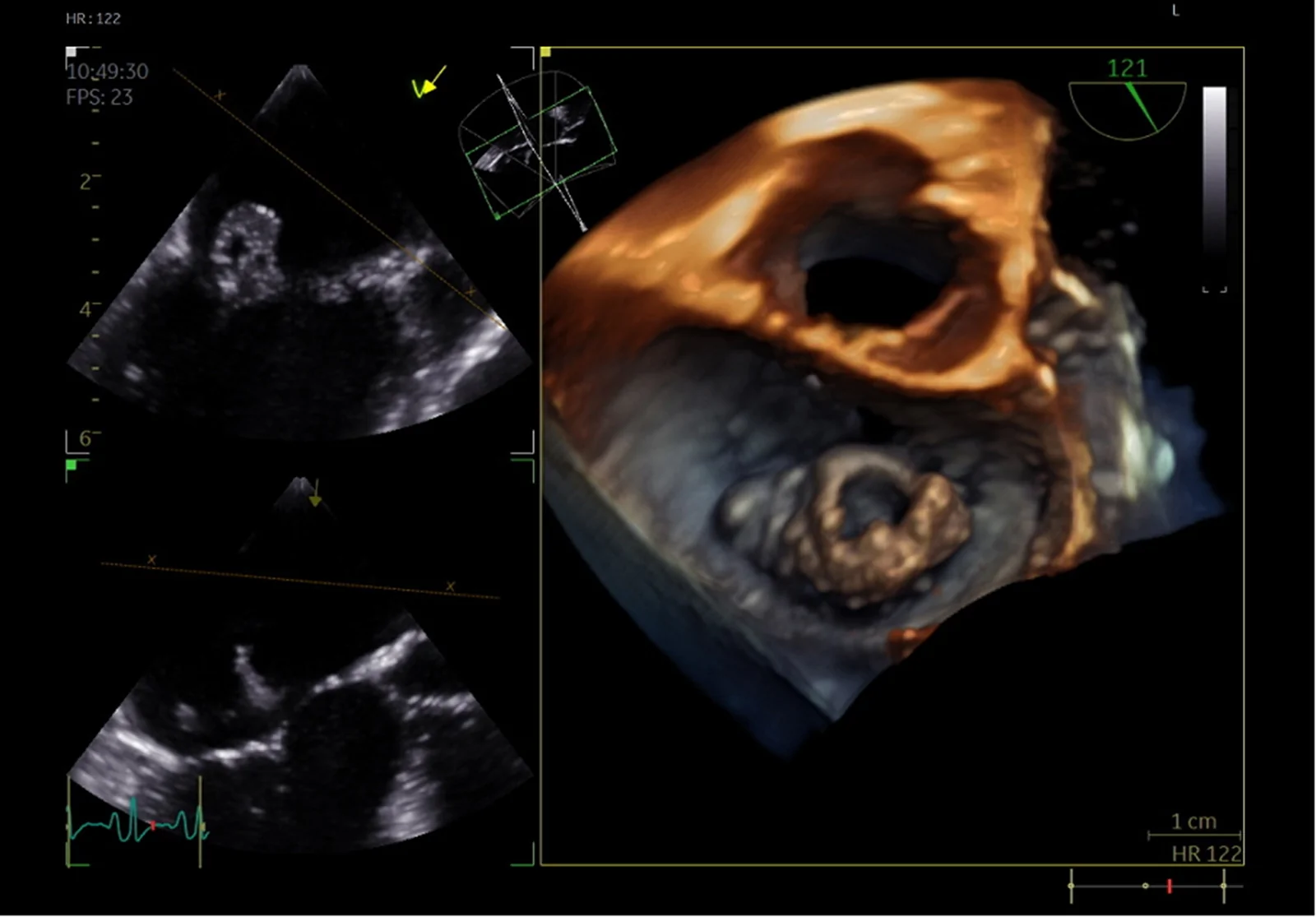
Transoesophageal echocardiography demonstrating large vegetation on A3 scallop of mitral valve. (Patient 2).

**Figure 3:**
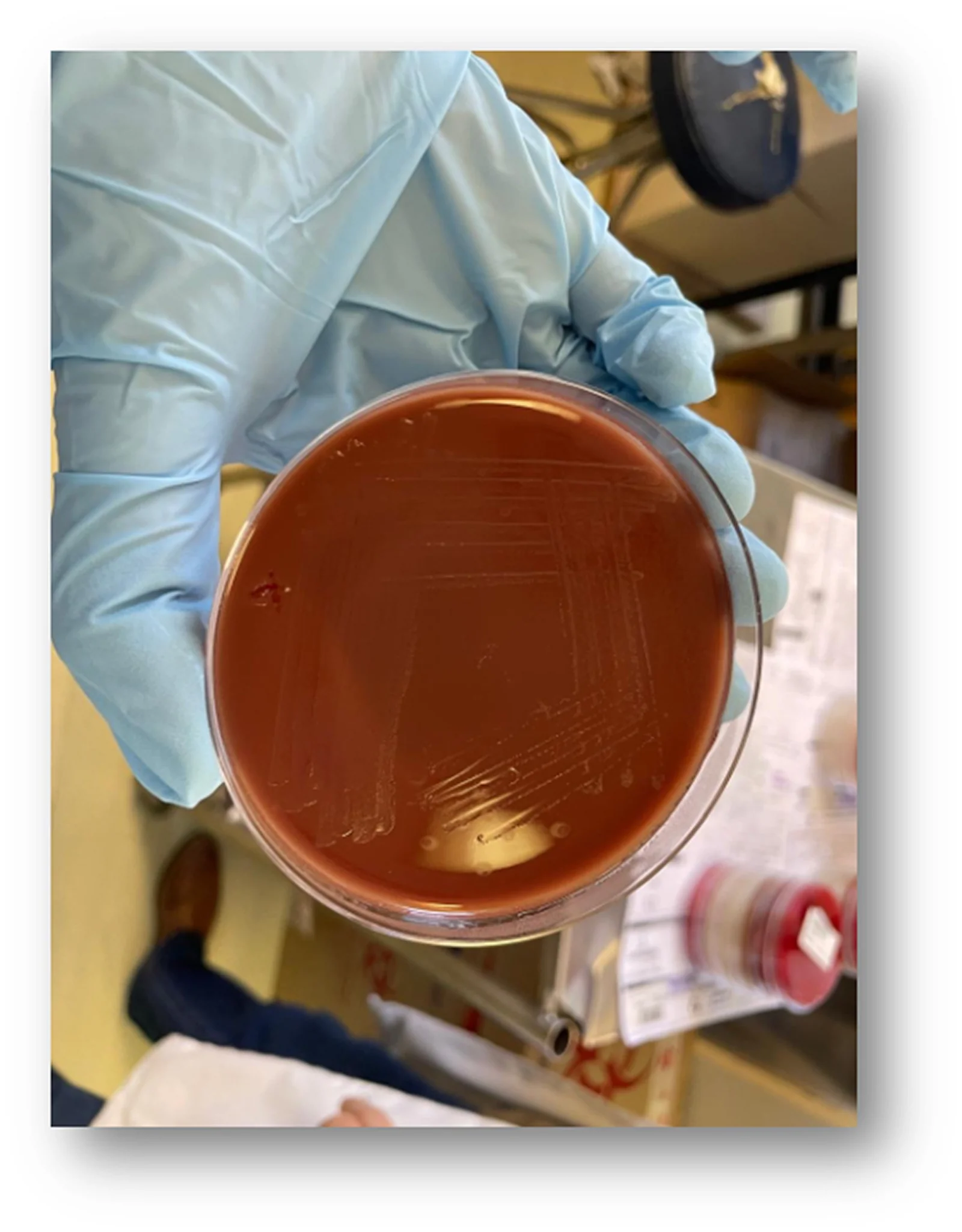
Gonococcal colonies grown on heat-treated chocolate agar enriched with haemin and NAD, subcultured after Gram-negative diplococci were seen on the positive blood culture (Patient 2).

Echocardiography revealed a large vegetation on the anterior mitral valve leaflet with an oscillating mass. Acute severe MR with a flail anterior segment was demonstrated, with preserved LV function (*Figure 2*). There was 2+ proteinuria on urine dipstick. Three aerobic blood culture bottles from a single antecubital site were obtained; one became positive at 14 h. When the Gram stain flagged Gram-negative diplococci, the broth was subcultured onto heat-treated chocolate agar enriched with haemin and NAD, yielding cephalosporin-susceptible *N. gonorrhoeae* (*Figure 3*). HIV testing was negative; rapid plasma reagin (RPR) was reactive, and following infectious diseases consultation the patient received a single intramuscular dose of benzathine penicillin G (2.4 million units) for asymptomatic early latent syphilis. The institutional empiric native-valve regimen included gentamicin, but this was withheld owing to renal dysfunction; ceftriaxone 1 g intravenously twice daily was therefore commenced, with doxycycline 100 mg orally twice daily added and later stopped as with previous patient. Targeted ceftriaxone 2 g intravenously daily (administered as 1 g twice daily) was continued for 6 weeks postoperatively. Emergency surgery was undertaken the day after admission (within 24 h), the 2023 ESC indication being acute severe MR causing refractory pulmonary oedema despite optimal medical therapy.

**Figure 4:**
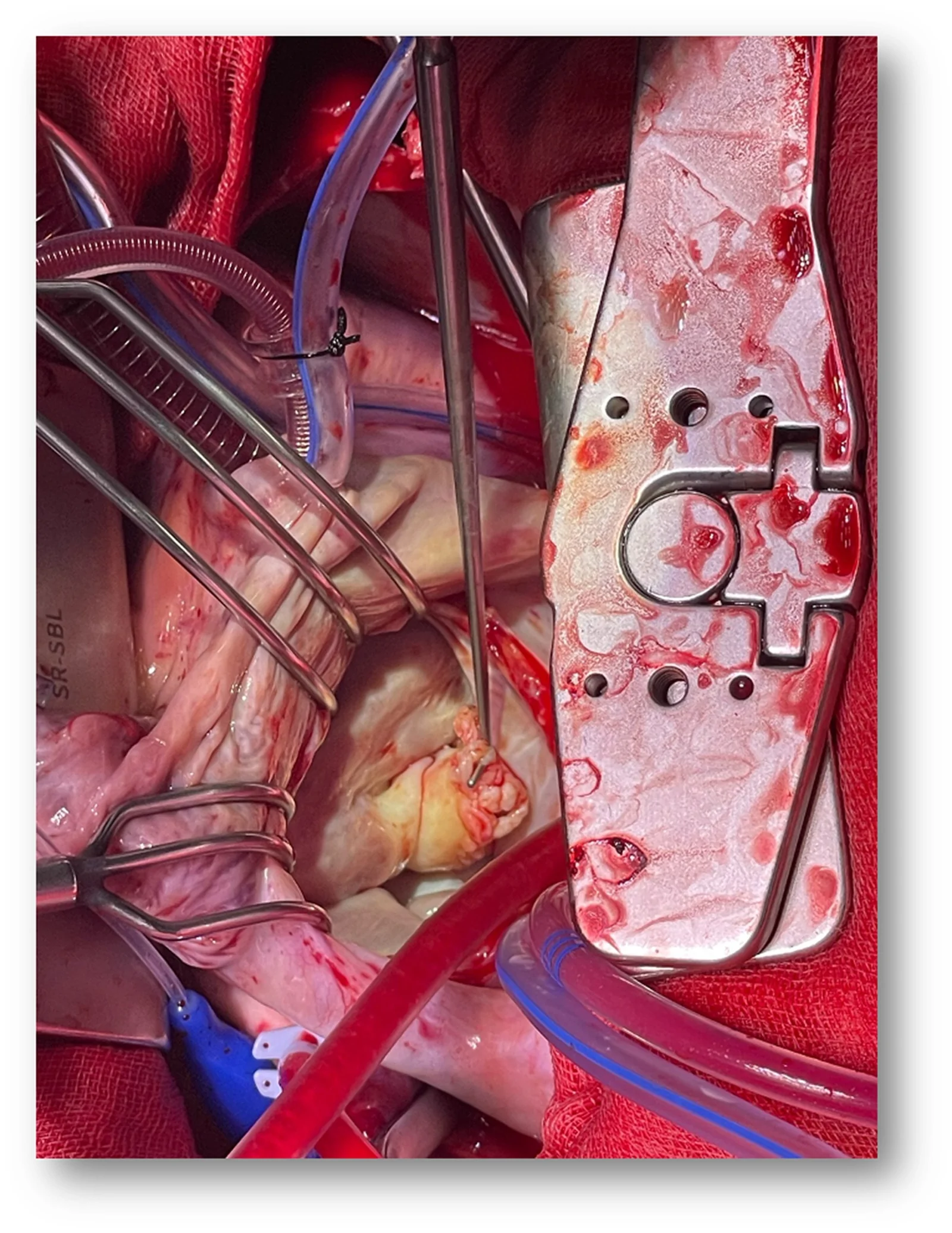
Intra-operative photograph with valve hook demonstrating a flail segment of the anterior mitral leaflet with attached vegetation, due to ruptured A2 chords (Patient 2).

Surgical findings were a large anterior leaflet vegetation with cord rupture (*Figure 4*) and a posterior annular abscess at P2–P3. A complex repair was performed: saphenous vein augmentation of the anterior leaflet free edge, a previously described technique,^4^ anchored by four Gore-Tex loops from the posterior papillary muscle, with an autologous pericardial strip for posterior annuloplasty.

Intra-operative valve tissue culture was positive for *N. gonorrhoeae*, while valve tissue PCR yielded no growth. The patient was discharged on warfarin following post-operative atrial fibrillation requiring cardioversion. This case met the definite IE Pathological Criteria (culture-positive valve), with a surgical Major Criterion from operative evidence of endocarditis (vegetation, leaflet flail, and an annular abscess).

All scheduled follow-up was defaulted. Approximately 9 months post-operatively he presented with a decreased level of consciousness and warfarin toxicity (INR >6), and died from a massive haemorrhagic stroke confirmed on computed tomography.

### Patient 3

A previously well 32-year-old woman presented with a 3-day history of dyspnoea, weakness, and fever (tachycardic, 40°C), on a background of 2 months of malaise and intermittent tender, swollen joints.

A pansystolic murmur radiating to the axilla and a raised jugular venous pressure were noted, with no peripheral stigmata of endocarditis.

Bedside echocardiography demonstrated a mobile lesion on the posterior mitral leaflet with associated MR.

An empiric culture-negative endocarditis regimen of benzylpenicillin (penicillin G) 5 million units 6-hourly, cefazolin 2 g intravenously three times daily, and gentamicin 120 mg intravenously daily was initiated after a single blood culture set was collected.

**Figure 5:**
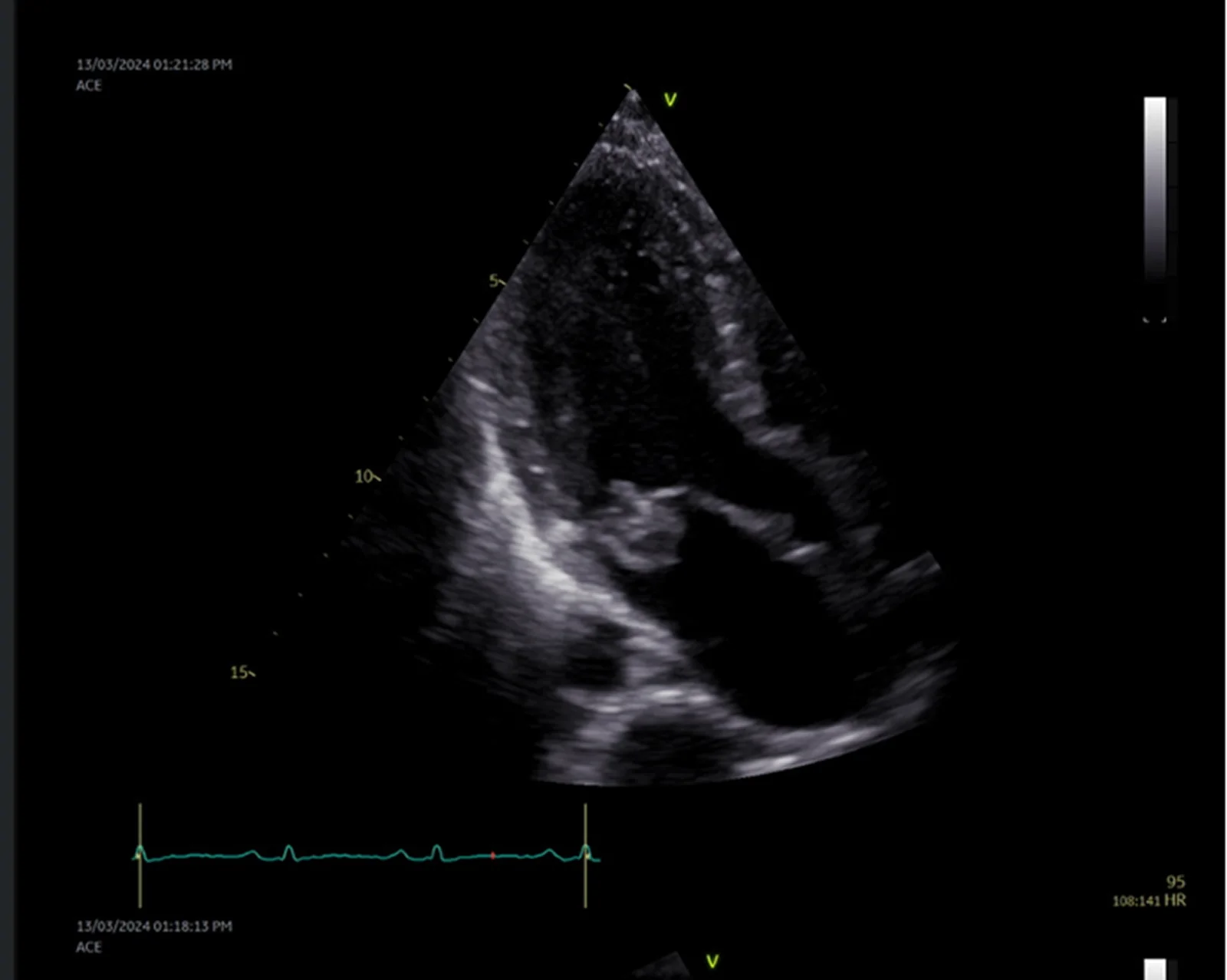
Apical three-chamber view via transthoracic echocardiography demonstrating anterior mitral valve perforation with a small vegetation on the atrial side (Patient 3).

Formal transthoracic and transoesophageal echocardiography revealed normal left ventricular size and function. The posterior mitral leaflet was thickened with a windsock deformity, and the anterior leaflet showed an A2 perforation with a small atrial-side vegetation (*Figure 5*), with acute severe MR and pericardial effusion.

A further three blood culture sets (totalling four bottles; two aerobic and one anaerobic BacT/Alert FA Plus per set, from contralateral antecubital site) were obtained after antibiotic initiation as described above. After 30 h of incubation, one culture bottle became positive with gram-negative cocci visible on microscopy, later identified as *N. gonorrhoeae*. The organism was resistant to ciprofloxacin, but sensitive to ceftriaxone.

The patient underwent urgent MVR (St Jude Medical 29 mm mechanical valve) 5 days after admission, after most of the posterior and anterior leaflets had to be debrided and resected. The resected mitral valve tissue was sent for PCR, which was positive for *N. gonorrhoeae*. Tissue culture remained negative.

Following organism identification, targeted ceftriaxone 2 g intravenously daily (as 1 g twice daily) was given for 6 weeks. HIV and RPR testing were negative. Her sexual partner was counselled and empirically treated for urethritis/discharge syndrome per local provincial guidelines (azithromycin 1 g orally and ceftriaxone 250 mg intramuscularly, single doses). A subxiphoid drain was placed for a residual simple pericardial effusion, and she was discharged after completing antibiotics.

The patient was non-compliant with warfarin therapy. Approximately 17 months after surgery, echocardiography demonstrated severe prosthetic mitral stenosis with a stuck occluder confirmed on fluoroscopy; at redo-sternotomy the explanted prosthesis showed thrombus and early pannus, and was replaced with a 27 mm St Jude Medical mechanical valve. She was well 5 months later, in sinus rhythm with a well-functioning prosthesis. As with the other cases, this met the definite IE Pathological Criteria (PCR-positive valve) together with a surgical Major Criterion (intra-operative leaflet destruction with visible vegetation).

## Discussion

### Presentation and diagnosis

Identifying the causative organism in IE is critical to effective treatment. *Neisseria gonorrhoeae* must be considered, particularly in blood culture negative infective endocarditis (BCNIE) and even in the absence of STI history or systemic DGI features, mandating a protocolised diagnostic workup for fastidious organisms.

At our institution, Bartonella species has emerged as the predominant causative organism in BCNIE.^5^ In that study, 32% of cases yielded no organism by conventional culture, underscoring the need for serology and broad-range PCR. Blood PCR is not routinely performed at our institution.

DGI results from bacteraemia due to *N. gonorrhoeae*, a gram-negative diplococcus, and manifests as polyarthralgia, tenosynovitis, dermatitis, or purulent septic arthritis.

While gonococcal infection is relatively common (weighted local prevalence 4.6%,^6^ endocarditis secondary to DGI remains rare and carries a mortality of 19% despite appropriate treatment.^1^

Patients with gonococcal endocarditis typically present in acute heart failure secondary to valvular regurgitation, often with minimal features of active gonococcal infection—as reflected by all three cases in this series.

The non-specific presentation, often with negative blood cultures, reinforces the need for a protocolised diagnostic workup. The confirmatory modality differed in each case (Patient 1: valve PCR; Patient 2: blood and valve culture; Patient 3: blood culture and valve PCR), yet in all three the explanted valve satisfied the Pathological Criteria and operative inspection provided a surgical Major Criterion, as summarized in Venn Summary. This underscores that gonococcal endocarditis may evade blood-culture-based diagnosis entirely.

A protocolised approach is required for fastidious organism identification. The broad-range 16S rRNA PCR platform, as utilized in this series, and immunofluorescence-based techniques are recommended by the 2023 Duke-ISCVID criteria for BCNIE.^7^ Nucleic acid amplification testing, including rectal and pharyngeal swabs, was not performed, as it is unavailable outside funded research at our institution owing to cost; its adoption would strengthen diagnostic yield and resistance profiling. HIV and syphilis screening is, however, performed routinely.

Lifelong anticoagulation carries significant morbidity in young patients, making valve repair desirable where achievable. Repairability should be critically assessed on the pre-operative echocardiogram and re-evaluated at surgical interrogation of the valve; where destruction precludes a durable repair, replacement is the only option, as determined by the operating surgeon. Repair was not achievable in Patients 1 and 3 owing to extensive leaflet destruction and peri-annular extension.

The 2023 ESC Guidelines on Infective Endocarditis^8^ recommend adequate pre- and intra-operative tissue sampling for culture and histology. In BCNIE, serology for *Brucella* spp., *Coxiella burnetii*, and *Bartonella* spp. should be obtained, as well as mycobacterial testing given the high TB prevalence in our setting.

Empiric therapy followed a typical native-valve regimen of a beta-lactam with gentamicin, consistent with the 2023 ESC recommendations; such regimens are institution-dependent and frequently amended with infectious diseases input, for example when renal dysfunction precludes an aminoglycoside, as in Patient 2. Antimicrobial selection for *N. gonorrhoeae* infection depends on local susceptibility profiles and culture results. Susceptibility testing was performed by disc diffusion, which precludes MIC quantification and provides only categorical classification. The WHO recommends ceftriaxone and azithromycin as first-line agents, though many countries now favour ceftriaxone monotherapy given rising macrolide resistance.^9,10^ Local *N. gonorrhoeae* strains are predominantly cephalosporin sensitive.^11^ In Europe, decreased ceftriaxone susceptibility has been reported in up to 15% of isolates, compared with under 2% in North America.^12,13,14^ Multiplex PCR assays may detect antimicrobial-resistant strains and are useful in BCNE, first-line treatment failure, and areas with a higher resistance burden.^15^

## Conclusion

A high index of suspicion for gonococcal endocarditis must be maintained despite absent DGI features, and a standardized approach to organism identification employed in all cases of BCNIE.

Extensive tissue destruction frequently necessitates replacement; repair should nonetheless be attempted where surgically feasible, given the morbidity of lifelong anticoagulation in young patients. Ceftriaxone monotherapy was effective in our setting.

## Data Availability

The data underlying this article are available in the article. Any further anonymised data will be shared on reasonable request to the corresponding author.

## Lead author biography

Stefan Van Der Westhuizen, MD, graduated from the University of the Free State in 2016 and is completing his residency in Cardiothoracic Surgery at Tygerberg Hospital, Stellenbosch University. He has a keen interest in pursuing congenital cardiac surgery training. This picture was taken during a recent clinical rotation at Sahlgrenska University Hospital in Sweden.

## Notes

**Consent:** The authors confirm that written consent for submission and publication of case report including images and text has been obtained from all patients in line with COPE guidelines.
