# Correction: A case series of confirmed Neisseria gonorrhoeae native mitral valve infective endocarditis: presentation and management

**DOI:** 10.1093/ehjcr/ytag643

**Published:** 2026-09-02

This is a correction to: Stefan Van Der Westhuizen, Johan Koen, Jan Steyn, Danielle Meintjes, Scott Lee-Jones, Jacques Janson, Anton Frans Doubell, A case series of confirmed *Neisseria gonorrhoeae* native mitral valve infective endocarditis: presentation and management, *European Heart Journal - Case Reports*, Volume 10, Issue 7, July 2026, https://doi.org/10.1093/ehjcr/ytag531

The following change has been made to the originally published paper.

The last name of the fourth co-author has been corrected to read: “Danielle Meintjes”.

